# A Narrative of a Journey to London in 1814, or a Parallel of the [between] English and French Surgery, Preceded by Some Observations on the London Hospitals

**Published:** 1816-07

**Authors:** 


*Relation d'un Voyage fait a Londres en* 1814; *ou Parallele de la Chirurgie Angloise avec la Chirurgie Françoise, précédé de Considerations sur les Hopitaux de Londres.* Par Phillibert-Joseph Roux, Docteur en Chirurgie; Chirurgien-en-seconde de l'Hopital de la Charité; Membre de la Legion d'Honneur; Professeur d'Anatomie, de Physiologie, et de Chirurgie, &c. &c. &c. Octavo, pp. 368. A Paris, 1815.

*A Narrative of a Journey to London in* 1814, *or a Parallel of the* [*between*] *English and French Surgery, preceded by*

---

ment, commits a mistake; giving to two of them, titles nearly synonymous. The translator, aware of some inaccuracy, pauses not to investigate; but manfully dashes through the difficulty by naming only *five* of the *six* classes just previously announced. Now had he only explored a few pages farther, the precise nature of the error, and mode of rectification, would have been at once obvious. Rev.

*some Observations on the London Hospitals.* By PHILLIBERT JOSEPH ROUX, Doctor in Surgery, &c. &c. &c. Translated from the French. Octavo, pp. 296. London, 1816.

ABOUT the time that our countryman, Mr. Cross, was in Paris, examining the chirurgical institutions and actual state of education and practice of that metropolis, M. Roux, a Parisian surgeon, of acknowledged talent and celebrity, came over to London with a similar view of investigating the œconomy and peculiarities of our surgical system. The interesting "Sketches" of Mr. Cross have recently been reviewed by us.* We have now to analyse the more diffuse and elaborate "narrative" of the French traveller.

The whole work consists of a chapter, entitled, *Preliminary Considerations,* and two distinct parts. Of the first of these, *the Hospitals, and Surgical Education in London,* constitute the subject. The second comprehends a Review of the *Surgical Doctrine and Practice of the English*; and the matter which it contains, although destitute of any marked division, may be conveniently arranged under the following heads:—*Dressing, and mode of operating of the English Surgeons:* Wounds; Ulcers; Fractures; Dislocations; Fungus Hæmatodes; Aneurisms; Diseases of the Eye; Operation for Fistula in Ano; Chronic Venereal Enlargement of the Testis; and Cancer of the Penis: Diseases of the Urinary Passages; Operation of Lithotomy; Hernia; and Amputation. To this order, we shall rigidly adhere in our present analysis.

"A few celebrated Surgeons have flourished, at remote intervals, in Italy, in Germany, and Holland. Camper, not long since, filled Holland with his renown. Germany possessed Richter, Siebold, and Bruninghausen: and Italy, at this moment, exults proudly in her Scarpa. But French and English Surgery have been, from the revival of the art, and yet remain, the only rivals. Wiseman was, both with respect to the time in which he lived, and his contributions to surgery, the Paré of England. From this memorable æra, in the history of our science, the two nations have never ceased mutually to exhibit distinguished surgeons. It would be difficult to decide, whether the French or English have contributed most to the rapid progress

* See our Number for February last.

which the art has made during the lapse of the last, and commencement of the present, century."

Such is the opening of M. Roux's *preliminary chapter*. He then proceeds to trace a sort of rapid parallel between the surgeons of the two nations, from Cheselden to those of the present day, and to recount the discoveries and inventions for which the science is respectively indebted to them. That the decisions of a Frenchman upon a subject in which his national vanity is interested, should be perfectly impartial and correct, no one will imagine: and hence we find him employing every little artifice to filch from our scale, in order to make his own preponderate. Of this, a single exemplification will suffice. M. Roux, in the course of his parallel, has occasion to mention the mode of operating for external aneurism, by tying the diseased artery above the tumor; "of which," he adds, "Desault and John Hunter conceived, at the same time, the fortunate idea." This view of the subject, we boldly affirm to be completely erroneous; and by the testimony of his own countryman, Bichat, are we willing that the point at issue shall be determined. It is well known, that, in the year 1783, memorable for John Hunter's first operation on aneurism, Desault operated in a case of popliteal aneurism upon a new plan. But Bichat, his pupil and editor of his surgical writings, expressly declares that, in so doing, he adopted the *process of Anel.** Now Anel's method consisted in applying a ligature to the morbid artery *directly above the aneurismal sac;* and how greatly inferior is this to Hunter's plan, every one, who has correct notions of the pathology of aneurism, and the principle upon which the ligature operates, must be sufficiently aware. As well might M. Roux invest with all the honours of the splendid Harveian discovery those of the great man's predecessors or contemporaries who had caught a faint and distant glimpse of the pulmonary circulation. We should scarcely have thought it worth while to pause upon this subject, did not M. Roux talk so loudly of his "impartiality," and exemption from "national prejudices." After specifying the objects of his visit to London, our traveller complains that he found many strong prejudices existing among us, with regard to the management and surgical practice of the Parisian hospitals: and fortunate, says he, was it "for the honour of French surgery, that the English surgeons were visited by

---

* "Le procédé suivi par Anel." *Œuvres Chirurgicales de Desault, &c. Par Bichat.* Tome ii. page 568.—Rev.

one of us capable at once of both appreciating what was doing among them, and of inspiring sufficient confidence to awaken their curiosity as to what was going on in France." Whether his exertions to dissipate these prejudices, and clear French surgery from the "unjust" reproaches cast upon it by "some young English physicians," were successful, he presumes not to determine. The terms in which his reception by the British surgeons is mentioned, we feel particular pleasure in recording.

"From what I knew of the national pride of the English, greater, perhaps, than that of any other civilized people, and disseminated alike through all the classes of society; from the reports which had reached us of the pretensions of their surgeons to a great superiority, I felt very apprehensive of experiencing from them but a cold reception. On the contrary, my reception was most flattering and honourable. I have not only to express my sense of those attentions and civilities which politeness claims for a foreigner of the same profession, and of some notoriety,—attentions of which we, Frenchmen, are, perhaps, lavish: all sort of respect was shewn me by the London surgeons. I received from them marks of esteem, and even of deference, which I was far from expecting. They were kind enough to instruct me in every thing which I wished to acquire from them, and evinced the greatest interest to learn the different peculiarities of our surgical practice. They even went so far as to request that I would perform, before them, upon some of their patients, various operations, according to the methods most commonly employed among us. The proposal was too honourable to be rejected; nor can I more properly acknowledge the politeness of the English surgeons towards me, than by detailing some of these particulars of my residence among them."

The moxa is a topical remedy much employed in France, but little known in this island. M. Roux mentions its successful application upon a patient of Mr. Lawrence's, at St. Bartholomew's, affected with paralysis of the deltoid muscle. The following description of its employment in a second case, we deem to be worth transcribing.

"At Guy's hospital, one of those which I most frequented, on account of Mr. Astley Cooper, a man possessing uncommon knowledge and activity, and who is there practising surgery with the greatest reputation, I found several patients with white swellings of the knee, so circumstanced as to warrant the employment of moxa. Mr. Cooper requested me to apply it, in his presence, upon a young woman, who suffered very severe pain externally to the patella. Upon this point, I burned a somewhat large cylinder of cotton. So great and rapid was the diminution of pain in the part upon which the moxa had acted, that, some days after, the patient assured Mr. Cooper of her willingness to submit to repeated applications of the moxa, if they were deemed necessary for her cure. She even appeared desirous that another should be immediately used on the internal side of the joint. With this young woman, the disease was too far advanced to afford hope of a favourable termination. It would, probably, continue its course, though with diminished rapidity and pain. Experience had taught me that such alone would be the result of the application of moxa under these circumstances; and I announced it as almost certain. If the event have verified my prediction, the English surgeons will, doubtless, in future, feel less averse from the employment of moxa. And thus I shall, probably, have contributed to naturalize among them this energetic remedy. May I add, that the application of moxa is not so severe, but that many people endure it, for the first time, without any expression of very acute pain; and others, who appeared to suffer more, submit with little reluctance to its reiterated employment."

The remnant of this chapter is occupied by an enumeration of the more distinguished surgeons, now practising in the British metropolis, and a brief notice of some of their literary productions. M. Roux remarks, with evident feelings of amaze, the great number of able men by whom surgery is cultivated among us, and the spirit of liberality and freedom of communication, which so honourably signalize their conduct towards each other.

"These and other causes, doubtles, of a nature yet more powerful, conspire to multiply, in London, and probably throughout all England, the number of men eminent in surgery. Our art enjoys there great consideration. It is especially from the time of John Hunter that surgery has been thus respected, and acquired a rank at least equal to that of medicine."

That persons totally unconnected with the healing art, and even men of distinguished rank, should regularly frequent our theatres of anatomy and surgery, is a fact with the information of which M. Roux seems to be particularly struck. The English surgeons, he allows, evince great enthusiasm in the cultivation of the science. The sketch, which he exhibits, in support of this assertion, is both faithful and strongly characteristic.

"There are some hospitals in London, which I have never once visited without seeing the principal surgeons surrounded by others of that capital, or by practitioners from remote towns, whom business had brought to London—men already full of years and experience,—each solicitous to see and observe, and eager for the acquirement of fresh knowledge. Nothing of this is remarked by foreigners in France. Rarely, indeed, are our young physicians and surgeons, after their departure from the schools, observed to revisit the scene of their early instruction."

Part First. — *Hospitals, and Surgical Education in London.* In the perusal of this section of the work, we have been greviously disappointed. We expected to have found here a systematic enumeration of all the principal metropolitan hospitals, a view of their internal œconomy and regulations, some notice of the system of instruction which they respectively exhibit, of the different lectures which constitute that system, and of the celebrated Professors and Teachers who preside over it, with striking traits or anecdotes, characteristic of any peculiarities of doctrine or opinion by which they are distinguished. But objects of comparatively inferior interest* occupy our traveller's attention during the first part of the sixty pages which compose this chapter; and its close, with the exception of some judicious observations on the internal arrangement of our hospitals, which we shall presently notice, is engrossed by loose and impertinent disquisitions on the excellencies of the French medical institutions, and French superiority, and avowals of a most generous intention of "proposing ourselves to the English as a model and example." Be not alarmed, Reader! nor harass your brain with conjectures, to discover wherein England may profit by French example. We will explain:

In Paris, all the civil hospitals are united under one administration; so that each constitutes a link of the chain, —an organ of one comprehensive system. And every person wishing for admission, must, except in cases of accident, present himself at the board of a central committee, instituted for that purpose, where the nature of his malady is investigated; and he receives a ticket of admission into this or that hospital, according to his age, disease, or considerations of convenience from the vicinity of his ordinary residence. All the London hospitals, on the other hand, are utterly unconnected with, and independent of, each other; and the patients are admitted, on the recommendation of subscribers, to the establishment. Now, it is the wish of M. Roux, to see established among us, "the

* Much is here said about our naval and military hospitals at Greenwich and Chelsea, the Military Asylum, and the Egyptian Ophthalmy, which some years since, committed such ravages there. M. Roux is evidently sceptical as to the *contagious* nature of this affection; which, he asserts, has *not* been communicated in *France* by the soldiers returning from Egypt.

system upon which these numerous asylums are formed into one whole; the parts of which, perfectly connected and exactly proportioned, seemed to exist, and, in fact, exist only, for each other." "But no," adds our philosophizing traveller, "the utter independence upon each other, of the London hospitals, the existence of all as so many private establishments without relation or connecting tie, is too conformable to the taste and genius of the English nation ever to be overthrown. Such a system favours too much the disposition of individuals to wage incessant conflicts against the supreme authority, and render themselves, as much as possible, independent of it; and acquire, in all public concerns, rights, prerogatives, and influence, for which they are indebted, in some measure, only to themselves: it will subsist for ever."

And may it so subsist, if its fall can be purchased only by sacrifice or humiliation of that boisterous and intrepid spirit; at the very breath of which, we have so often known

"Oppression droop, and tyranny turn pale!"*

But we think that M. Roux had done well to abstain from all comment on our political principles. The scourge is certainly in our hands: pity, however, for a fallen and degraded rival disarms retaliation. To the plan upon which patients are, at present, admitted into the hospitals of London, as well as Paris, he objects, and not, perhaps, without reason.

"These hospitals," he observes, "should be accessible, without so many formalities, to the unfortunate requiring assistance; nor can they be too much exempted from all preparatory measures, commonly so little suited to their state of privation. It is in the hospitals themselves, and by a system of regulations rigorously enforced, that the abuses which might result from this plan (undue facilities of admission) should be obviated."

The remaining observations of M. Roux upon the London hospitals, regard either their number, size, architecture, and internal arrangement; or some peculiarities of their interior organization with respect to the distribution of the sick, and visits of the surgeons. Upon these observations, we shall dwell at some length, as constituting,

---

* Happy for France and humanity, had she, previously to the commencement of her troubles, possessed, and exerted, somewhat of this turbulent anti-despotic spirit!

in our opinion, one of the most sensible and instructive portions of the work.

Twenty-two hospitals, properly so called, exist in London. They are, none of them, so large as the Hotel-Dieu, of Paris; nor, on the other hand, so small as several of the institutions of that capital. They regularly contain between nine and ten thousand patients, double the number which the Parisian hospitals are capable of receiving. Few of them only were founded at the expence of government, or are supported by it. In this respect, they again differ from the hospitals of the rival metropolis.

The architecture and construction of the London hospitals are much superior to those of Paris, and attract the admiration of our traveller. Many of them may, for architectural elegance and simplicity, be deservedly ranked among the most remarkable monuments of a city, "truly poor in fine public edifices." Their dimensions are always sufficiently large for the number of patients which each is destined to accommodate; and, in point of internal arrangement, every advantage has been attained, and every convenience studied. The wards are rather small, but contain proportionately few patients. They are generally well-lighted, but almost invariably too low; and great attention is paid to cleanliness.

"But, with all this, the interior of their wards exhibits not the imposing spectacle by which most of ours are distinguished: for their beds are low, narrow, ill-furnished, destitute of curtains, or only having small ones; which, placed at the head of each, serve only as a paltry decoration. In our hospitals, on the contrary, the patients find a bed at least as good, and often better, than that which they have quitted; and it is pleasing to see them wide, sufficiently high, well-furnished, and surrounded with curtains, the least advantage of which consists in diminishing the nakedness of the wards; and which are, moreover, useful to the patients in protecting them from the impression of cold during the winter season, and affording them, in the day-time, a degree of obscurity favourable to the repose of which they may stand in need. Thus too, are they enabled to screen themselves from the view of those by whom they are immediately surrounded; and exclude the afflicting spectacle, so frequently displayed in hospitals, of persons in the agony of dissolution."

Rarely are more than two or three surgeons placed at the head of a Parisian hospital. Ours, on the other hand, have commonly four or six: and this, by M. Roux, is justly regarded as an advantage; but he remarks with surprise, that, even in our largest hospitals, the wards are indiscriminately appropriated to medical and surgical cases.

Of the disadvantages of this plan, or rather want of plan, he institutes an elaborate discussion. We shall endeavour to strip his arguments of all their characteristic and wearisome prolixity, and condense them within periods of somewhat less than half a page length each.

Surgical diseases, he contends, except during the prevalence of hospital gangrene, cause less impurity of air than internal maladies: for noxious effluvia are more copiously extricated from the subjects of the latter, than the miasmata from wounds and ulcers. The mortality is incomparably greater among the sick than among the wounded: and it is of importance that the minds of those, who have sustained severe injuries or operations, should not be agitated by scenes of death, or other painful impressions. Such patients, moreover, stand in need of perfect tranquillity, especially during the night, when sleep should obliterate the remembrance of their sufferings. But this is denied them, while surrounded by the victims of internal disease. Restlessness, indeed, is produced by many surgical maladies; but patients, in this state, are rarely heard to complain. Silence, therefore, generally reigns at night in the surgical, but not in the medical wards, where the groans of those in pain, the cough of the consumptive, the incoherent ravings of the delirious, and the presence of nocturnal attendants, break in upon the stillness of the hour, and dispel sleep. Thus are the surgical patients, in our hospitals, unnecessarily exposed to the influence of physical and moral causes, alike injurious. Nor is their presence among the sick, less productive of unpleasant consequences to the latter. The great concourse of students assembled at the hour of dressing, the frequent spectacle of dreadful accidents and operations, and the noise inseparable from a surgical ward, where many of the patients are able to go about and converse with each other, must greatly disturb the comfort and repose of such as are affected by internal diseases.

"I am persuaded," continues M. Roux, "that in the hospitals where the two departments are confounded, in those of London for example, the physicians' patients dread greatly the hour appointed for the surgeons' visit."

The policy of this separation is, we believe felt and acknowledged by many of our surgeons and physicians. An attempt has even been made to introduce it into one of the London hospitals, but hitherto without success.

Another defect equally prejudicial, in M. Roux's opinion, to the patient and the science, is the practice which our Physicians and Surgeons have adopted of visiting the Hospitals, committed to their direction, only twice or thrice a week, instead of doing it every day, as is the custom in Paris. Vainly may it be urged that, in every hospital, the Physician has Assistants, to whom, under the name of Apothecaries, the preparation of the medicines, and task of daily visiting the sick, are confided. Must it not be injurious to the patient to be treated alternately by two men, who, perhaps, differ widely in their theoretic and practical views; and of whom one may be compelled to follow implicitly the blunders of the other. Nor are the interests of the science itself less deeply implicated: for, although many internal diseases are so tardily developed that we may delineate them correctly, without watching their daily progress; there are yet others which, from the rapidity of their march and frequent variation of character, require constant attention; and for a precise knowledge of which, the physician should rely solely upon his own observation — see with his own eyes. Surgical cases, it is true, are less subject to irregularity and variation: hence the daily visits which the French hospital-surgeons have imposed upon themselves may be deemed superfluous. But surgery, like medicine, has its acute diseases; and surgical affections, perhaps more frequently than those of the other class, demand the most prompt and efficient succour. "If I may judge by my own feelings and experience," adds M. Roux,

"A zealous surgeon at the head of a large hospital, finds daily occasion to observe something which he would not have seen on the morrow, yet regret to have missed; and constantly discovers something to be done which he would not wish to see executed by an assistant,"

With respect to the hour at which our surgeons and physicians make their visit to the hospitals, the observations of our traveller are, perhaps, less striking, and his arguments less conclusive. The exacerbations, crises, and variations of internal diseases commonly take place during the night; and should be observed, as soon as possible, after their occurrence. Morning too is the time for prescribing changes of food and medicine. In surgical maladies, this is of less importance; except with patients upon whom some surgical operation is about to be performed; and here attention to preserve the mind from the noxious influence of moral causes, requires that the visit of the surgeon be made early in a morning.

"It is impossible, in our hospitals to keep a patient ignorant of the day of operation: frequently it is appointed by himself. The day arrives: the patient has passed a tranquil night; undisturbed by dread of the approaching operation. [This we much doubt.] But if this operation be deferred till mid-day, the imagination, from the moment of awaking, will be haunted by some terrific idea. If, on the other hand, a patient, during the night which precedes the operation, have been restless and disturbed by gloomy presentiments, humanity impels us to relieve him promptly from a situation thus distressing."—Again. "Many operations are followed by primary accidents, as hæmorrhage and spasm: and, if either take place, it is commonly within twenty-four hours, but seldom very soon, after the operation. Suppose then that it has been performed at mid-day; and that consecutive hæmorrhage occurs: most probably, the bleeding will happen during the night: while, after operations performed in a morning, such an accident, if occurring, will almost invariably show itself from noon to close of day. And it is sufficiently evident that, in all respects, night is a time the least propitious for remedying such accident."

Yet there is one point of view under which M. Roux has not considered, and indeed could scarcely be expected to consider, this subject; since he sojourned but one month among us, and that not during the busy season of the London schools. Deeply are engraven on our remembrance the feelings of fretfulness and regret, with which, upon some hasty summons from "Sir Charles," we have often been compelled to quit an interesting piece of dissection, or forego the advantages of a luminous demonstration, to follow the great man, at a hand-gallop, through the hospital wards; and, in less than an hour, hurry to anatomical lecture, breathless and exhausted, and scarcely retaining a faint and confused recollection of what we had seen, heard, or executed. The regular business of the London schools rarely commences, in the theatre and dissecting-room, before ten o'clock! And what an œconomy of time would it be to the student,—what an admirable improvement of those hours too commonly lost in sleep, or shamelessly squandered in beautifying and perfuming his person; if the visits of the physicians and surgeons were made, in a morning, at seven or eight o'clock. The advantages of such system to the practitioners themselves, men generally and most deservedly high in the public estimation, and whose more private duties would thus suffer little interruption; the benefits resulting from it to the unfortunate objects of their care, and the cause of public instruction, are incalculable; and would amply compensate for any additional exertion which, at first, the practice might demand. Look at the aged Pelletan, paying his morning visit to the *Hotel-Dieu* by candle-light, in the depth of a rigorous winter.* This, to our eye, is a spectacle far more "imposing" than all the "spacious wards" of the French hospitals; than all their "curtained couches" can exhibit; a scene, to us, inexpressibly fine—we had almost said—affecting; and fraught with touches of the morally sublime! Such are the men to whom, in our opinion, the character of "enthusiasm for the science" is truly, and most correctly, applicable. May we pause, for a moment to offer the zealous veteran our expressions of veneration and respect!

The Sketch given by M. Roux of the *Surgical Education in London* is extremely limited and imperfect, as, perhaps, might be expected from the period and duration of his visit among us. After noticing our College of Surgeons as an institution greatly inferior to the *Facultés de Medécine,* of Paris, he states it, as an "extraordinary circumstance" that, of all the branches of the science, comparative anatomy is there taught with the *greatest* minuteness and attention. How our traveller gained this intelligence, we know not; but certainly the surprise he felt on receiving it, will be equalled by that which its repetition must excite in those of our countrymen; who have daily occasion to observe and regret, that comparative anatomy has *not* yet acquired, among us, that high consideration, or been cultivated with that ardour, which, if we contemplate only the light occasionally elicited from it upon the structure and functions of the *human* organs, it is, doubtless, eminently calculated to inspire. The anatomical museum of the college, and that of Messrs. Wilson and Bell in Windmill Street, are mentioned by M. Roux in terms of high and merited eulogy. The former, he thinks, is less a museum of surgical, than comparative anatomy, in consequence of the great number of preparations taken from the inferior classes of the animal kingdom which it contains. In the facility of prosecuting anatomical studies, London possesses, at present, a decided superiority to Paris; but the latter boasts a regular system of clinical lecturing to which our metropolis is unfortunately a stranger. Here follow some remarks upon the discouragement of private anatomical instruction in Paris; which,

---

* *See* Mr. Cross's interesting "Sketches." Page 64.

as we profess not clearly to comprehend, we shall decline attempting to analyse.

The lectures in London, he is informed, are very concisely delivered; while those of the Parisian teachers are more diffuse, or, as the Englishmen in general consider it, needlessly prolix.

"But, perhaps, these two modes of instruction, equally faulty, are adapted to the peculiar turn of mind of the men for whom they are chosen. A serious reflecting character, prone to meditation, and conspicuous for its early developement, eminently distinguishes the English nation. The Englishman, while yet young, is remarkable for a certain maturity of reason and judgment; which, when the instruction of any science is in view, allows one to reckon as much on the operation of his thought as on the simple exercise of his memory. With equal capacity for mental exertion, for the culture of the sciences, and the inspirations of genius, the French youth is more impetuous and volatile: his reasoning powers are more slowly developed; and, when he enters upon the studies of science, it is necessary, at least for a time, to cultivate exclusively his memory, and leave little to his reflection. There exists, perhaps, also in Frenchmen, a necessity of diffusing, of communicating to others, in full detail, the knowledge we have acquired. The observation of Seneca, that he would be content with ignorance, if he were forbidden to communicate his acquirements to other men, expresses correctly one of the features of our character."

Thus concludes the first Part of M. Roux's narrative.

As the reader will probably, by this time, begin to think, like ourselves, that he has swallowed enough of our French *Eau medicinale* for one dose; we shall here desist, and reserve the remnant for our next visit. Far, however, be from us the wicked insinuation that we have discovered, in the *chirurgical potion* of M. Roux, the *insipid* properties of an *aqueous* fluid; nor indeed mark we any thing of its *simplicity*. For his verily is a composition of the *polypharmaceutic* school; and the ethereal spirit, which it contains, greatly deteriorated in its operation by the admixture of heterogenous matter; yet withal very largely diluted! Its *analysis* will be terminated in our next number.

